# Association Between Hormone Replacement Therapy and Development of Endometrial Cancer: Results From a Prospective US Cohort Study

**DOI:** 10.3389/fmed.2021.802959

**Published:** 2022-01-17

**Authors:** Ying Liang, Haoyan Jiao, Lingbo Qu, Hao Liu

**Affiliations:** ^1^Department of Pharmacy, Guangdong Food and Drug Vocational College, Guangzhou, China; ^2^Department of Chemistry, Zhengzhou University, Zhengzhou, China

**Keywords:** hormone replacement therapy (HRT), endometrial cancer (EC), body mass index, estrogen, progesterone

## Abstract

Although hormone replacement therapy (HRT) use is associated with elevated endometrial cancer(EC) risk, little evidence assesses potential effect-modifiers on HRT-related EC in a long-term follow-up. In this large-scale longitudinal cohort study, we tried to evaluate the association between different HRT types/methods use and risk of EC, and reveal this risk within different body mass index (BMI) groups. In whole cohort, 677 EC occurred during mean 11.6 years follow-up. Cox proportional hazards regression was used to estimate multivariable-adjusted hazards ratios (HRs) and 95% confidence intervals (CIs) with HRT status (never, former, or current) for risk of EC incidence. Current HRT use was not significantly associated with EC risk (HR for current vs. never HRT use: 1.13; 95% CI: 0.92, 1.38) in the whole cohort, but presented a dose-response effect on increased EC risk (HR for >10-year use vs. never HRT use: 1.73; 95% CI: 1.35, 2.21). Moreover, EC risk differed in distinct regimens or subsets (all *P*_interaction_ < 0.05). Estrogen-only use was associated with elevated EC risk (HR for current vs. never HRT use: 1.51; 95% CI: 1.12, 2.04), but women with high BMI (> 30 kg/m^2^) who currently use estrogen-only harbored decreased EC risk (HR: 0.56; 95% CI: 0.38, 0.82) compared to counterparts without HRT use. Estrogen-only use is associated with increased EC risk, and precise monitoring of EC development for postmenopausal women with long-term HRT use are urgently needed. BMI could serve as an important surrogate to assess this risk.

## Introduction

Population aging is increasing worldwide. In 2020, the number of women aged 50 years and older were up to 69 million, and by 2050, women above 50 are projected to total to 90 million (1). The mean age of natural menopause is 49 years (2). Most perimenopausal women would suffer from a series of menopause symptoms such as hot flashes, night sweats, vaginal dryness, and low mood or anxiety, which may persist for a decade or longer (3). In addition, up to 84% of postmenopausal women experience genitourinary symptoms, such as vulvovaginal atrophy and incontinence (4). The burden of menopausal symptoms can considerably affect the personal, social, and work lives of women (3). Hormone replacement therapy (HRT) is the most effective treatment for such vasomotor and genitourinary symptoms (5). In high-income countries, there were about 600 million woman-years of HRT use in the period 1970–2019, and about 12 million users in the 2010s, of whom 6 million were in the United States and the United Kingdom alone (6, 7).

Nevertheless, there is increasing attention to possible health effects of HRT beyond alleviation of menopausal symptoms, such as secondary cancer risks. Concerns of subsequent risks of gynecological tumors are raised from postmenopausal women who take HRT (8), like cervical squamous cell carcinoma (9) and endometrial cancer (EC) (10). Endometrial cancer (EC) is the most common female gynecological malignancy with an estimated lifetime incidence of 4% (11). The most characteristic pathophysiological feature of EC is its hormone-dependence. Options of HRT typically include unopposed estrogen, combined therapy (estrogen and progesterone), and tibolone. Unopposed estrogen use increases the risk of endometrial hyperplasia (12), and risk of EC is directly associated with circulating estrogen and androgen levels (13). However, progestogens are considered as an inhibitor of carcinogenesis and endometrial tumor suppressor (14, 15). An umbrella review indicated that the level of evidence from epidemiology studies on the association between HRT and risk of EC is weak because of insufficient observations (16). The counterbalancing effect of HRT is dependent on the duration of HRT. For example, the Women's Health Initiative trial indicated that daily use of a synthetic progestin, in combination with estradiol, over 5 years significantly decreases the risk of EC (17). In addition, body mass index (BMI) might interact with the effect of HRT on EC (18, 19). Specifically, obesity-related EC risk, with a relative risk of 1.59 per 5 kg/m^2^ incremental increase (20), could be explained by a hyperestrogenic state, which is caused by higher rates of conversion of androgenic precursors to estradiol in adipose tissue (18, 21). Postmenopausal women on estrogen-containing HRT are in an excess estrogen state.

The aim of this study was to further investigate the effect of different types and durations of HRT on EC development, with focus on interaction between BMI and HRT on the effect of EC risk.

## Methods

### Participants

The longitudinal data analyzed in this study were obtained from the PLCO trial (https://cdas.cancer.gov/plco/), which is a large-scale randomized trial designed and sponsored by the National Cancer Institute (NCI) to determine the effects of screening on cancer-related mortality and secondary endpoints in men and women aged 55–74, and includes following five ClinicalTrials.gov registration numbers: NCT00002540 (prostate), NCT01696968 (lung), NCT01696981 (colorectal), NCT01696994 (ovarian), and NCT00339495 (EEMS). Approximately 155,000 participants were enrolled between November 1993 and July 2001, and cancer data were collected up to December 31, 2009. In this study, 78,209 female participants were randomly assigned to the intervention arm (*n* = 39,103) and the control arm (*n* = 39,106). We further excluded 33,006 female participants if they underwent hysterectomy before the trial started (*n* = 27,575), did not return baseline questionnaires (*n* = 3,001); had cancer history before completing supplemental questionnaire (SQX) that recorded HRT regimens (*n* = 5,188); or had less than 6 months follow-up after SQX completion or without follow-up data (*n* = 133). Finally, 45,203 women with follow-up data remained in the cohort (Supplementary Figure S1).

The study protocol of the PLCO Cancer Screening Trial was approved by the Institutional Review Board of the National Cancer Institute. All the participants provided written informed consent. The data used in this study had been approved, and the project ID was PLCO-734. Given that the PLCO trial provided de-identified information of patients, the Guangdong Food and Drug Vocational College Institutional Review Board considers PLCO data analyses to be exempt from Institutional Review Board review.

### Outcome Ascertainment

Outcomes included diagnosis of EC and time to EC events. All reports of EC were followed up, and medical records were abstracted and reviewed for case ascertainment. We extracted EC cases according to International Classification of Diseases for Oncology, Third Edition (ICD-3, which was coded as C54.1).

### Exposure Ascertainment

At baseline, the women were asked whether they had ever used HRT (choices included current, former, and never use). On subsequent questionnaires (SQX), if they had used HRT since the previous questionnaire, they would further indicate HRT type, method of HRT use, as well as the duration of use during that time period (choose included estrogen only, progesterone only, estrogen + progesterone combined; pills or cream; and <1 year, 1–3 years, 3–5 years, 5–10 years, and >10 years).

### Covariates

The women reported their age at baseline/menarche/menopause, history of fertility, race (white, non-Hispanic Black, non-Hispanic, Hispanic Asian, and others), education level (less than high school, high school graduate or equivalent, post high school education, college education, or higher), marital status (singled, married, divorced/separated, or widowed), birth control pill use, and family history of EC. Data on BMI (kg/m^2^), physical activity, and income (per month) (< $20,000, $20,000–49,000, $50,000–99,000, $100,000–200,000, or > $200,000) were collected at baseline and updated on subsequent follow-up questionnaires (SQX).

### Statistical Analysis

We classified eligible women into three groups according to HRT use status (never, former, and current). To present the baseline characteristics across HRT status, mean (interquartile range, IQR) for continuous variables that are normally distributed as indicated by Shapiro-Wilk normality test (all *P* < 0.05) and frequencies (percentages) for categorical variables were calculated. *T*-test and chi-square test were performed to test for differences between continuous and categorical, respectively, participant characteristics.

Cox proportional risk regression was conducted to calculate crude and adjusted hazard ratios (HRs) and 95% confidence intervals (CIs), where the proportional hazard (PH) assumption was examined by Schoenfeld residual test (22). Confounding factors were screened using the strategy “change-in-estimate” (23) to assess if they were associated with both HRT use and EC risk or they changed the crude risk estimate by >10% in bivariate analyses. Time-to-EC was defined as days from DQX completion to EC diagnosis. Censoring time for EC incidence models was calculated as days from DQX completion to death or last contact occurring on or before 31 December 2009 among participants without EC.

Furthermore, cross-products of important co-variates with HRT use in the multivariable-adjusted model were tested by Likelihood ratio tests, and *P*_interaction_ < 0.05 were considered as a cut-point of significant effect modification. Accordingly, subgroup analyses stratified by crucial variables that were mentioned by prior literature (24), such as BMI (20–25, 25–30, and >30 kg/m^2^), and HRT method (pills and cream), were performed.

*P* values reported (two-sided) to be <0.05 were considered statistically significant. All the analyses were conducted using the R software (version 4.0.1).

## Results

In total, 45,203 eligible women were included in this study, 18,286 in the “never” group, 7,826 in the “former” group, and 19,091 in the “current” group. Their baseline characteristics are shown in Table 1. Compared with the women who never used HRT, the current users were younger, earlier to have menopause, better educated, and were more likely White, married, and to perform regular exercise and use birth control pills, but are less likely to have a family history of EC.

**Table 1:** Baseline characteristics of female subjects by status of hormone replacement therapy (HRT) in the PLCO screening trial.

	**HRT status**			***P***
	**Never**	**Former**	**Current**	
**Number of participants**	18,286	7,826	19,091	
	**Median (IQR)**	**Median (IQR)**	**Median (IQR)**	
**Age, years**	73 (67–77)	72 (67–77)	68 (65–73)	**<0.001** ^**a**^
**Pack-years of cigarette**	0 (0–19)	0 (0–22)	0 (0–19.5)	**<0.001** ^**a**^
	**N (%)** ^**b**^	**N (%)** ^**b**^	**N (%)** ^**b**^	
**Trial arm**				
Intervention	9,076 (49.6)	3,943 (50.4)	9,577 (50.2)	0.44 ^b^
Control	9,210 (50.4)	3,883 (49.6)	9514 (49.8)	
**Race/Ethnicity**				
White, non-Hispanic	15,858 (86.7)	6,948 (88.8)	1,7445 (91.4)	**<0.001** ^**b**^
Black, non-Hispanic	1,360 (7.4)	407 (5.2)	426 (2.2)	
Hispanic	277 (1.5)	94 (1.2)	237 (1.2)	
Asian	625 (3.4)	317 (4.1)	877 (4.6)	
Other^c^	158 (0.9)	58 (0.7)	102 (0.5)	
Unknown	8 (0.0)	2 (0.0)	4 (0.0)	
**Marital status**				
Single^d^	897 (4.9)	263 (3.4)	647 (3.4)	**<0.001** ^**b**^
Married	11,825 (64.7)	5,299 (67.7)	14,060 (73.6)	
Divorced or separated	2,315 (12.7)	1,119 (14.3)	2,565 (13.4)	
Widowed	3,214 (17.6)	1,134 (14.5)	1,803 (9.4)	
Unknown	35 (0.2)	11 (0.1)	16 (0.1)	
**BMI, kg/m** ^**2**^				
<18.5	189 (1.0)	84 (1.1)	222 (1.2)	**<0.001** ^**b**^
18.5–25	4,163 (22.8)	1,933 (24.7)	6,529 (34.2)	
25–30	4,045 (22.1)	1,813 (23.2)	4,798 (25.1)	
>30	3,103 (17.0)	1,286 (16.4)	2,616 (13.7)	
Unknown	6,786 (37.1)	2,710 (34.6)	4,926 (25.8)	
**Income, per month**				
< $20,000	2,146 (11.7)	733 (9.4)	1,068 (5.6)	**<0.001** ^**b**^
$20,000–49,000	4,551 (24.9)	1,999 (25.5)	4,829 (25.3)	
$50,000–99,000	2,047 (11.2)	1,083 (13.8)	4,138 (21.7)	
$100,000–200,000	462 (2.5)	240 (3.1)	1,217 (6.4)	
>$200,000	64 (0.3)	42 (0.5)	246 (1.3)	
Unknown	9,016 (49.3)	3,729 (47.6)	7,593 (39.8)	
**Education level**				
Less than high school	1,542 (8.4)	530 (6.8)	517 (2.7)	**<0.001** ^**b**^
High school graduate or equivalent	5,825 (31.9)	2,141 (27.4)	4,115 (21.6)	
Post-high school education	2,317 (12.7)	1,027 (13.1)	2,248 (11.8)	
College education or higher	8,556 (46.8)	4,113 (52.6)	12,184 (63.8)	
Unknown	46 (0.3)	15 (0.2)	27 (0.1)	
**Age at menarche**				
<10	261 (1.4)	111 (1.4)	244 (1.3)	**0.1** ^**b**^
10–11	3,268 (17.9)	1,354 (17.3)	3,333 (17.5)	
12–13	9,687 (53.0)	4,212 (53.8)	10,569 (55.4)	
14–15	4,115 (22.5)	1,771 (22.6)	4,090 (21.4)	
≥16	917 (5.0)	362 (4.6)	819 (4.3)	
Unknown	38 (0.2)	16 (0.2)	36 (0.2)	
**Age at menopause**				
<40	433 (2.4)	178 (2.3)	270 (1.4)	**0.001** ^**b**^
40–44	1,659 (9.1)	724 (9.3)	1,201 (6.3)	
45–49	4,808 (26.3)	2,015 (25.7)	4,096 (21.5)	
50–54	9,008 (49.3)	3,820 (48.8)	9,279 (48.6)	
≥55	2,267 (12.4)	1,067 (13.6)	3,917 (20.5)	
Unknown	111 (0.6)	22 (0.3)	328 (1.7)	
**History of fertility**				
No	1,611 (8.8)	594 (7.6)	1,661 (8.7)	**0.02** ^**b**^
Yes	16,654 (91.1)	7,223 (92.3)	17,406 (91.2)	
Unknown	21 (0.1)	9 (0.1)	24 (0.1)	
**Physical activity**				
Active less than one time per month	1,643 (9.0)	664 (8.5)	1,409 (7.4)	**<0.001** ^**b**^
Active at least one time per month	9,227 (50.5)	4,226 (54.0)	12,361 (64.7)	
Unknown	7,416 (40.6)	2,936 (37.5)	5,321 (27.9)	
**Birth control pills**				
No	10,470 (57.3)	3,640 (46.5)	6,984 (36.6)	**<0.001** ^**b**^
Yes	7,788 (42.6)	4,182 (53.4)	12,099 (63.4)	
Unknown	28 (0.2)	4 (0.1)	8 (0.0)	
**Family history of endometrial cancer**				
No	17,448 (95.4)	7,477 (95.5)	18,281 (95.8)	**<0.001** ^**b**^
Yes	482 (2.6)	220 (2.8)	527 (2.8)	
Possible	(2.0)	(1.7)	(1.4)	

a *P value was calculated from Kruskal–Wallis test*.

b *P value was calculated from Chi-Square test*.

c *Other race including Pacific Islander and American Indian*.

d *Single including never married*.

*IQR, interquartile range*.

*Bold values denote statistical significance at the p < 0.05 level*.

A total of 677 EC events occurred during mean 11.6 years follow-up (Table 2), 267 in never group (13/10,000 person-years), 110 (12/10,000 person-years) in former group, and 300 (14/10,000 person-years) in current group. After adjustment for age at baseline, age at menopause, BMI, education level, race, physical activity, family history of EC, and birth control pills, current HRT use was significantly associated with increased EC risk (HR for current vs. never HRT use: 1.13; 95% CI: 0.92, 1.38; HR for >10-year use vs. never HRT use: 1.73; 95% CI: 1.35, 2.21, Table 2). Furthermore, EC risk differed in different HRT regimens, namely, estrogen-only, progesterone-only, and estrogen plus progesterone combined. Estrogen-only use was associated with elevated EC risk (HR for current vs. never HRT use: 1.51; 95% CI: 1.12, 2.04; HR for >10-year use vs. never HRT use: 2.92; 95% CI: 2.06, 4.14, Table 2), whereas no relationship between progesterone-only or estrogen plus progesterone combination use and risk of EC was found in status or time accumulated comparisons (Table 2).

**Table 2:** HRs (95% CIs) for endometrial cancer incidence and death by hormone replacement therapy status in the screening arm of the PLCO screening trial: 1993–2009.

**HRT**	**No. of cases**	**Person-years**	**Incidence rate/10,000 person-years**	**Hazard ratio (95% confidence interval)^c^**		
				**Unadjusted model**	**Adjusted model 1^**a**^**	**Adjusted model 2^**b**^**
**OVERALL**						
**HRT status**						
Never	267	212,470	13	1.00 (reference)	1.00 (reference)	1.00 (reference)
Former	110	91,671	12	0.96 (0.77, 1.19)	1.03 (0.79, 1.33)	1.07 (0.83, 1.39)
Current	300	221,757	14	1.08 (0.91, 1.27)	1.08 (0.89, 1.32)	1.13 (0.92, 1.38)
**HRT duration**						
Never	267	212,470	13	1.00 (reference)	1.00 (reference)	1.00 (reference)
<1 years	71	69,468	10	0.81 (0.63, 1.06)	0.83 (0.61, 1.13)	0.85 (0.62, 1.15)
1–3 years	67	58,118	12	0.92 (0.70, 1.20)	0.86 (0.62, 1.19)	0.86 (0.62, 1.19)
3–5 years	55	48,166	11	0.91 (0.68, 1.22)	0.91 (0.65, 1.28)	0.93 (0.66, 1.30)
5–10 years	83	64,882	13	1.02 (0.80, 1.30)	1.08 (0.81, 1.42)	1.13 (0.85, 1.51)
>10 years	132	71,148	19	**1.48 (1.20, 1.82)**	**1.53 (1.21, 1.95)**	**1.73 (1.35, 2.21)**
**P**_**trend**_				0.003	0.002	<0.001
**ESTROGEN ONLY**						
**HRT status**						
Never	267	212,470	13	1.00 (reference)	1.00 (reference)	1.00 (reference)
Former	22	14,490	15	1.21 (0.78, 1.87)	1.25 (0.80, 1.95)	1.35 (0.86, 2.10)
Current	60	34,444	17	**1.39 (1.05, 1.83)**	**1.39 (1.04, 1.86)**	**1.51 (1.12, 2.04)**
**HRT duration**						
Never	267	212,470	13	1.00 (reference)	1.00 (reference)	1.00 (reference)
<1 years	9	8,153	11	0.88 (0.45, 1.71)	0.89 (0.45, 1.73)	0.91 (0.47, 1.78)
1–3 years	5	9,327	5	0.43 (0.18, 1.04)	0.43 (0.17, 1.03)	0.45 (0.19, 1.11)
3–5 years	12	8,137	15	1.17 (0.66, 2.09)	1.15 (0.64, 2.07)	1.21 (0.67, 2.18)
5–10 years	16	10,065	16	1.26 (0.76, 2.09)	1.24 (0.74, 2.07)	1.37 (0.82, 2.29)
>10 years	40	13,120	31	**2.43 (1.74, 3.39)**	**2.54 (1.80, 3.58)**	**2.92 (2.06, 4.14)**
**P**_**trend**_				**<0.001**	**<0.001**	**<0.001**
**PROGESTERONE ONLY**						
**HRT status**						
Never	267	212,470	13	1.00 (reference)	1.00 (reference)	1.00 (reference)
Former	3	2,382	16	1.00 (0.32, 3.12)	0.97 (0.31, 3.05)	0.98 (0.31, 3.09)
Current	12	7,985	15	1.19 (0.67, 2.13)	1.15 (0.64, 2.07)	1.24 (0.69, 2.25)
**HRT duration**						
Never	267	212,470	13	1.00 (reference)	1.00 (reference)	1.00 (reference)
<1 years	0	1,741	0	/	/	/
1–3 years	4	2,105	19	1.51 (0.56, 4.05)	1.42 (0.53, 3.85)	1.34 (0.49, 3.63)
3–5 years	2	1,528	13	1.04 (0.26, 4.18)	0.98 (0.24, 3.96)	1.05 (0.26, 4.26)
5–10 years	4	2,481	16	1.28 (0.48, 3.43)	1.23 (0.46, 3.32)	1.32 (0.49, 3.57)
>10 years	5	2,489	20	1.59 (0.66, 3.86)	1.61 (0.66, 3.92)	1.88 (0.77, 4.59)
**P**_**trend**_				0.29	0.33	0.20
**ESTROGEN PLUS PROGESTERONE COMBINED**						
**HRT status**						
Never	267	212,470	13	1.00 (reference)	1.00 (reference)	1.00 (reference)
Former	17	13,479	13	1.00 (0.61, 1.64)	0.99 (0.60, 1.64)	1.04 (0.63, 1.72)
Current	103	91,095	11	0.90 (0.72, 1.13)	0.88 (0.68, 1.13)	0.93 (0.72, 1.21)
**HRT duration**						
Never	267	212,470	13	1.00 (reference)	1.00 (reference)	1.00 (reference)
<1 years	9	11,714	8	0.61 (0.31, 1.19)	0.59 (0.30, 1.16)	1.20 (0.83, 1.74)
1–3 years	26	19,555	13	1.06 (0.71, 1.59)	1.01 (0.66, 1.54)	1.04 (0.71, 1.52)
3–5 years	17	19,481	9	0.70 (0.43, 1.14)	0.66 (0.40, 1.10)	0.69 (0.41, 1.14)
5–10 years	34	27,132	13	1.00 (0.70, 1.43)	0.97 (0.67, 1.40)	1.00 (0.65, 1.53)
>10 years	34	26,321	13	1.03 (0.72, 1.47)	1.04 (0.72, 1.51)	0.59 (0.30, 1.16)
**P**_**trend**_				0.80	0.85	0.60

a *Adjusted for age (continuous)*.

b *Adjusted for age (continuous), age at menopause (<45, ≥45), body mass index (BMI) (continues), education (less than high school, high school graduate or equivalent, post-high school education, college education or higher), race (white, black, others including Hispanic, Asian, Pacific islander), physical activity (active less than one time per month, active at least one time per month), family history of endometrial cancer (no, yes), birth control pills (no, yes)*.

c *Cox proportional hazards regression*.

*HRT, hormone replacement therapy; BMI, body mass index; PLCO, Prostate, Lung, Colorectal, and Ovarian Cancer. Bold values denote statistical significance at the p < 0.05 level*.

The subgroup analyses for association between estrogen-only use and EC risk, were conducted by stratifying BMI and the HRT method, which was changed across each subgroup (all *P*_interaction_ < 0.05, Table 3). Interestingly, the increased risk of EC from the estrogen-only regimen observed before reduced among the women who used estrogen creams (HR: 1.59; 95% CI: 1.02, 2.48, Table 3) compared with those using pills (HR: 2.23; 95% CI: 1.53, 3.26, Table 3). Obese women who currently use estrogen only harbored decreased EC risk (HR: 0.56; 95% CI: 0.38, 0.82, Table 3) compared to counterparts without HRT use, but in the normal/underweighted subgroup (<25 kg/m^2^), and estrogen-only use was associated with increased risk of EC (HR for current vs. never HRT use: 2.75; 95% CI: 1.79, 4.24, Table 3).

**Table 3:** Risk of endometrial cancer incidence from estrogen-only use stratified by body mass index, hormone replacement therapy method in the PLCO Cancer Screening Trial: 1993–2009.

**Characteristics**	**Never^**a**^**	**Former**	**Current**	** ${\text{P}}_{\text{interaction}}^{\text{c}}$ **
**BMI, kg/m** ^**2**^				
<25 (n)	4,352	2,017	6,751	**<0.001**
Breast cancer cases (n)	28	14	112	
Multivariable adjusted HR (95% CI)^b^	1.00 (reference)	1.11 (0.58, 2.12)	**2.75 (1.79, 4.24)**	
25–30 (n)	4,045	1,813	4,798	
Breast cancer cases (n)	50	26	68	
Multivariable adjusted HR (95% CI)^b^	1.00 (reference)	1.23 (0.77, 1.98)	1.31 (0.89, 1.92)	
>30 (n)	3,103	1,286	2,616	
Breast cancer cases (n)	89	37	40	
Multivariable adjusted HR (95% CI)^b^	1.00 (reference)	1.05 (0.71, 1.55)	**0.56 (0.38, 0.82)**	
**HRT method**				
Pills (n)	5,056	3,265	11,885	**0.009**
Breast cancer cases (n)	35	44	175	
Multivariable adjusted HR (95% CI)^b^	1.00 (reference)	**1.98 (1.27, 3.10)**	**2.23 (1.53, 3.26)**	
Cream (n)	3,723	1,257	2,061	
Breast cancer cases (n)	48	12	38	
Multivariable adjusted HR (95% CI)^b^	1.00 (reference)	0.77 (0.41, 1.46)	**1.59 (1.02, 2.48)**	

a *Women who did not receive HRT was considered as reference*.

b *Adjusted for age (continuous), age at menopause (<45, ≥45), body mass index (BMI) (continuous), education (less than high school, high school graduate or equivalent, post-high school education, college education or higher), race (white, black, others including Hispanic, Asian, Pacific islander), physical activity (active less than one time per month, active at least one time per month), family history of endometrial cancer (no, yes), birth control pills (no, yes). HRT, hormone replacement therapy; PLCO, Prostate, Lung, Colorectal, and Ovarian Cancer; Q, quintile*.

c *P_interaction_ was calculated by adding the cross-product of HRT status and BMI/HRT method in the Multivariable-adjusted COX proportional hazards regression model*.

*Bold values denote statistical significance at the p < 0.05 level*.

## Discussion

In this longitudinal study, our results found that estrogen-only use for HRT was associated with increased risk of EC development, and an obesity paradox that this association was completely reversed in obese women was confirmed. Furthermore, women with long-term use (over 10 years) of HRT, especially on the type of estrogen only, or with estrogen pills had significantly higher risk of EC.

An epidemiological study demonstrated that less than 25 and 38% of women with EC are diagnosed before menopause in Western and Asian countries, respectively (25). A meta-analysis including 28 studies (10), indicated that estrogen alone, tibolone and sequential combined therapy increase the risk of EC, even when treatment lasts less than 5 years, but continuous combined therapy might provide a lower risk than never use, and therapy for more than 10 years does not increase risk. Our study had a comparable follow-up with that of postmenopausal Breast Cancer Detection Demonstration Project (26), but did not find association between short-term use of estrogen only (<5 years) and increased risk of EC because of limited samples size and statistical power.

It is well-established that the endometrium is extremely sensitive to unopposed estrogen, either endogenous or exogenous, which can induce endometrial hyperplasia. Although simple hyperplasia is rarely directly transformed to EC, it can evolve to atypical hyperplasia, which is a precancerous lesion. Thus, progesterone is the important hormone to maintain a eutrophic endometrium, and is mainly used for women with preserved uterus. Its role is to overcome the proliferative effect of estradiol and induce differentiation of the glands, stroma, and vessels of the endometrium (27). A large amount of evidence shows that continuous-combined therapy with synthetic progestins reduces the risk of EC (17, 28–30). Combining HRT with natural progestins would increase the risk of EC when dosage is lesser and duration is longer (31, 32). In contrast, many studies support that HRT with estrogens only increases the risk of developing EC (28–30, 32, 33). This is consistent with what we found, namely, the carcinogenic effect of estrogens could be alleviated by progestins. Additionally, estrogen type and duration of use are significant modifiers on the association between estrogen use and EC risk. Long-term use of estrogens might augment the harmful effect, and short-term use of ≤ 5 years is recommended. Previous studies have also indicated that the risk was directly proportional to the duration of sequential therapy (26, 32, 34). Although both estrogen pills and cream increased the risk of EC, the effect tended to be obvious when women choose pills. It could be explained by the direct association between circulating estrogen and androgen levels and carcinogenesis of EC (13). Additionally, the reduced EC risk from estrogen-only cream compared with that administrated with pills is due to different metabolic processes (35). Therefore, doctors give greater consideration to transdermal HRT, in line with the NICE guideline (36).

Body mass index (BMI) is a risk factor for EC, and the association of BMI with cancer risk ranks highest for EC (20, 37). However, this study interestingly found an “obesity paradox” when comparing the risk between current estrogen users and women who never use estrogens. It seems that obese women bear lower HRT-related EC risk. As we know, non-exposed HRT women with high BMI are associated with increased EC risk than their underweight counterparts (16), leading to shrinking the “riskgap” in EC development between obese women with HRT exposure. Pathophysiologically, cancer cachexia and biological mechanisms such as differences in body composition and adiposity, and nutritional reserve to face anti-cancer treatments, somewhat contribute to this obesity paradox (38, 39). In the future, research should focus on the standardized incidence ratio of EC from HRT use, which is compared with the general population.

The prospective cohort design, comprehensive assessment of HRT status and types, and long follow-up period are the strengths of this study. Several limitations should also be noted. First, the PLCO trial only recruits participants aged 55–74 years. Thus, the results of this study cannot be extended to other age groups. Second, women who were diagnosed with EC before SQX completion or have invalid SQX response were excluded in this study, which might induce biases. Previous studies have indicated that most HRT-associated ECs are, thus, endometrioid adenocarcinomas (32, 33, 40), but we did not acquire the histologic subtype of EC in this PCLO project and failed to assess this relationship.

## Conclusion

Long-term use (> 10 years) of estrogen-progesterone combined with HRT increased the risk of EC development. Specifically, HRT with estrogens only significantly increases the risk of EC, but there were no associations of HRT with progesterone only. The carcinogenic effect of estrogen is more obvious in pills than in cream, and among underweight women than obese women.

## Data Availability Statement

The original contributions presented in the study are included in the article/Supplementary Materials, further inquiries can be directed to the corresponding author/s.

## Ethics Statement

The studies involving human participants were reviewed and approved by Institutional Review Board of the National Cancer Institute. The patients/participants provided their written informed consent to participate in this study.

## Author Contributions

HL designed the study and collected data. YL, HJ, and LQ analyzed the data. YL wrote the article. All authors reviewed and approved the final version of the manuscript.

## Funding

This research was funded by the Characteristic Innovative Project of Guangdong Province Ordinary Institution of Higher Education (2020KTSCX259) and Science and Technology Program of Guangzhou (202102080546).

## Conflict of Interest

The authors declare that the research was conducted in the absence of any commercial or financial relationships that could be construed as a potential conflict of interest.

## Publisher's Note

All claims expressed in this article are solely those of the authors and do not necessarily represent those of their affiliated organizations, or those of the publisher, the editors and the reviewers. Any product that may be evaluated in this article, or claim that may be made by its manufacturer, is not guaranteed or endorsed by the publisher.

## Supplementary Material

Supplementary Figure S1 Study flowchart for identifying eligible individuals. PLCO, the Prostate, Lung, Colorectal and Ovarian Cancer Screening Trial; SQX, supplemental questionnaire.

## Abbreviations

PLCO: the Prostate, Lung, Colorectal and Ovarian Cancer Screening Trial
EC: endometrial cancer
HRT: hormone replacement therapy
SQX: supplemental questionnaire
IQR: interquartile range
HRs: hazards ratios
CIs: confidence intervals.
