# Supplementary figures and images for: Association Between Hormone Replacement Therapy and Development of Endometrial Cancer: Results From a Prospective US Cohort Study
Source: Front Med (Lausanne). 2022 Jan 17;8:802959. doi: 10.3389/fmed.2021.802959 (PMC8801732; doi:10.3389/fmed.2021.802959)

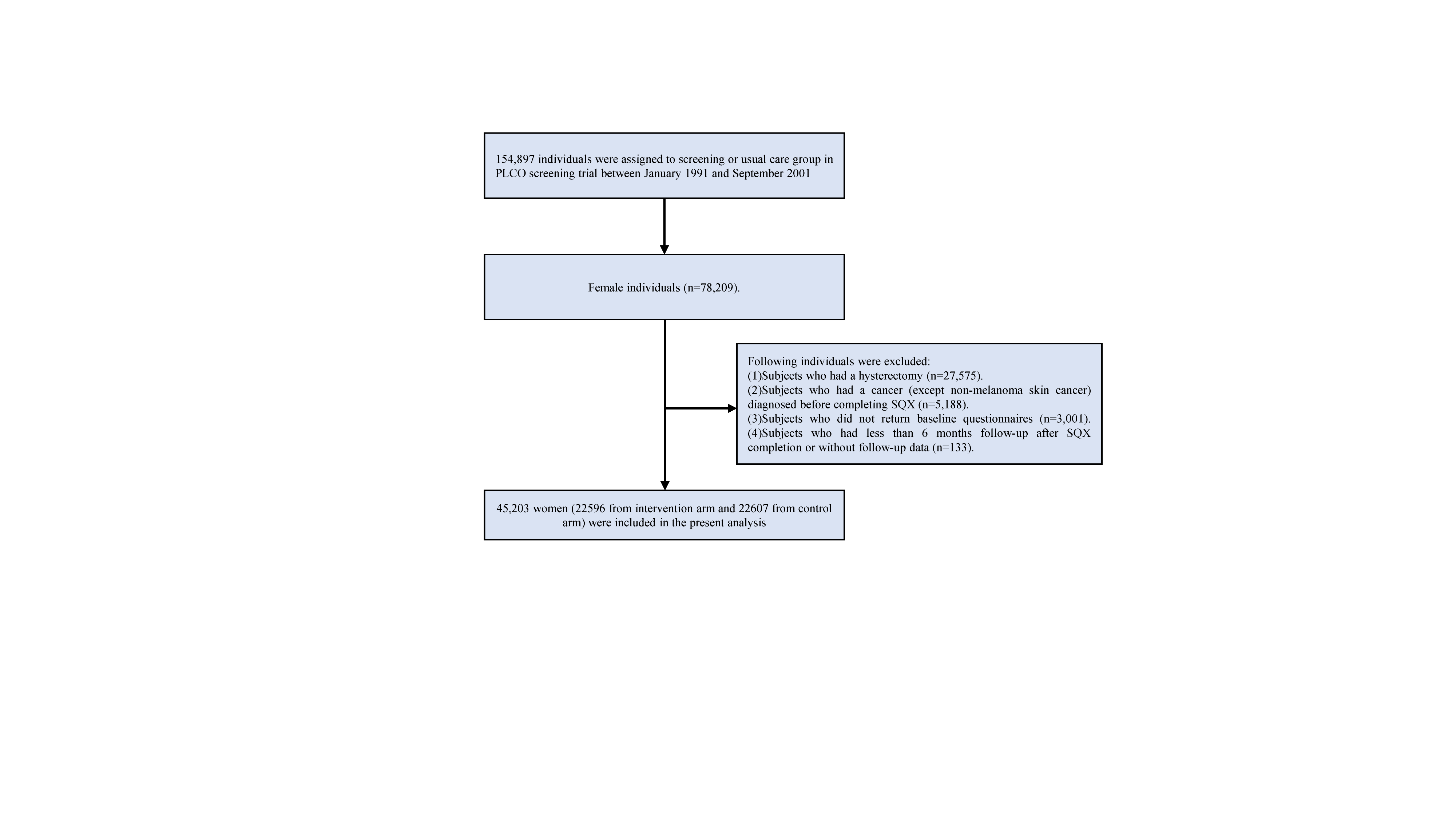

Supplement: Supplementary Figure S1 — Study flowchart for identifying eligible individuals. PLCO, the Prostate, Lung, Colorectal and Ovarian Cancer Screening Trial; SQX, supplemental questionnaire. [file Image_1.TIF]
